# Editorial: Early detection of neurodegenerative disorders using behavioral markers and new technologies: New methods and perspectives

**DOI:** 10.3389/fnagi.2023.1149886

**Published:** 2023-02-28

**Authors:** Valeria Manera, Erika Rovini, Peter Wais

**Affiliations:** ^1^CoBTeK Lab, Department of Speech Therapy, Université Cote d'Azur, Nice, France; ^2^Department of Industrial Engineering, University of Florence, Florence, Italy; ^3^Department of Neurology, Neuroscape and Weill Institute for Neurosciences, University of California, San Francisco, San Francisco, CA, United States

**Keywords:** neurodegenerative diseases, behavioral markers, new technologies (ICT), artificial intelligence, early assessment, Parkinson's disease (PD), Alzheimer's disease, mild cognitive impairment

Neurodegenerative disorders (ND) are a common cause of mortality and morbidity worldwide, particularly in older people. As life spans continue to increase, the incidence of neurodegenerative diseases is expected to increase as well [World Health Organization (éd.), 2017]. In addition to pathological threats, adults above 60 years show increasing vulnerability to broad decline in memory, attention, and multi-tasking as a matter of normal aging. Indeed, cognitive impairment, with or without ND, can predict imminent motor decline or neuropsychiatric symptoms, such as apathy and depression.

ND, such as Alzheimer's Disease (AD) and Parkinson's Disease (PD), develop progressively over many years. After an asymptomatic stage (only revealed by biomarker evidence), cognitive and neuropsychiatric symptoms start to appear and then worsen over time, until they lead to a loss of autonomy in activities of daily living. Multi-domain interventions (targeting simultaneously multiple areas, such as cognition, lifestyle, and physical activity) are showing promising results in delaying ND progression (Kivipelto et al., 2018; Meng et al., 2022). The earlier and more personalized the intervention, the more promising the results are hypothesized to be (Devos et al., 2021; Solomon et al., 2021; Röhr et al., 2022). For this reason, detecting ND in its early stages is an important clinical and research challenge.

Biomarkers can predict risk for AD, PD, and other ND, several years before the clinical symptoms appear. For instance, biomarkers of amyloid β pathology (low CSF Aβ42 or increased CSF Aβ40–Aβ42 ratio; increased tracer retention in amyloid PET) and biomarkers of tau pathology (increased phosphorylated tau in CSF; increased tracer retention in tau PET) can characterize AD pathology before the appearance of clinical symptoms (Dubois et al., 2021). Also, α-synuclein species, lysosomal enzymes, markers of amyloid and tau pathology, and neurofilament light chain biomarkers from CSF and blood reflect the pathophysiology of Parkinson's disease and are providing promising preliminary results for the early diagnosis (Parnetti et al., 2019). Furthermore, low baseline Aβ42 in the CSF of non-demented PD patients predicts development of cognitive impairment over time (Leaver and Poston, 2015). However, lumbar puncture and PET imaging are invasive, expensive, and realized only in specialized clinical settings. For these reasons, there is growing interest in finding new approaches that can be employed to investigate early signs of cognitive, motor, and behavioral decline that can be indicative of potential ND, and can thus help to identify people who should be tested with the more specialized biomarker indices. In order to be helpful, these technological approaches must be non-invasive, rapid to administer and safe to apply outside of specialized clinics. Basic research with wearable sensors, new Information and Communication Technologies (such as automated video and audio-analyses), Virtual Reality video games, smartphone or tablet applications and olfactory tests have recently shown promise to reveal changes in subjects' abilities and behaviors, which in turn can support the clinician in early identification of subtle disorders (Robert et al., 2016; Maremmani et al., 2018).

In this Research Topic we collected nine papers that examined specific non-invasive tools, methods or technologies applied in assessments of cognitive and/or motor performance, or neuropsychiatric symptoms, in cohorts of older adults with ND or with diminished cognitive function.

## Subjective reports

Pang et al. showed the interest of validated subjective cognitive complaints for dementia screening. Specifically, in a large sample of community-dwelling older adults, they demonstrated that combining a reliable single-question assessment for subjective cognitive decline with an objective tool (such as the Montreal Cognitive Assessment battery-MoCA, Nasreddine et al., 2005) can efficiently discriminate dementia patients from healthy older adults in the community, suggesting the potential of self-reports for large-scale screenings.

## Neuroimaging and psychophysiological measures

In terms of PD screening, Chang et al. showed that resting state EEG characteristics extracted by Holo-Hilbert Spectral Analysis and processed with machine learning algorithms are important markers for the diagnosis of PD, in particular showing a reduction of β bands in frontal and central regions, and reduction of γ bands in central, parietal, and temporal regions in PD patients. Also, these characteristics are positively correlated with the depression severity (i.e., θ and β bands values in all brain regions). Similarly, Ma et al. found that the incidence of multiple step saccades in their visually guided reactive saccade task could be a complementary biomarker for the early diagnosis of PD. This approach provides for an easy assessment of ND through eye-tracking.

## Kinematics

Assessing motor performance and action kinematics is relevant not only for PD detection, but can be employed also to screen for mild cognitive impairment, especially in dual-task conditions. Ali et al. found that kinematic gait parameters of knee peak extension angle during a dual task performance (walking + story recall) were sensitive enough to discriminate individuals with MCI from healthy controls.

Measuring the global level of motor activity and sleep using actigraphy recorded over 7 days, Cai et al. found that diurnal vector magnitude and total time in bed correlated negatively with apathy severity in patients with Cerebral Small Vessels Disease. This confirms that objective motor indicators can be relevant also for the assessment of neuropsychiatric symptoms, such as apathy and depression.

## Brain oxygenation and autonomic biomarkers

Other non-invasive biomarkers are also starting to show promising results. For instance, Li et al. showed that objective indicators of brain oxygenation status and cerebral autoregulation function (assessed using near-infrared spectroscopy technology and a non-invasive blood pressure device) can reflect cognitive function, and correlate with the level of cognitive decline in older adults assessed using the MoCa.

In their review, Barthelemy et al. highlighted the importance of monitoring autonomic biomarkers (such as heart rate variability) parameters, in addition to classical cardiovascular risk factors, to increase the prediction of stroke.

## Genetics and plasma biomarkers

Tung et al. confirmed the interest of combining genetic (Apolipoprotein E-ApoE polimorfism) and clinical information to detect cognitive decline and optimize interventions. Interestingly, across comorbidities, functional gastrointestinal disorder was the strongest predicting factor for dementia in ε4 allele carriers.

Finally, Liang et al. investigated the predictive ability of preoperative plasma biomarkers along with cerebral oxygen saturation for the incidence of post-operative cognitive dysfunction in older patients with MCI. They drafted recommendations on the cerebral oxygen saturation level based on Aβ-42 status to reduce the risk of post-operative cognitive disfunction.

Taken together, these results corroborate the idea that new technologies, non-invasive sensors, and machine learning algorithms can complement and support traditional assessment, and their results converge with traditional biomarker evidence. The important developments from this topic should motivate near term research to examine which markers type of screening should be employed for a patient depending the type of risk factors. Furthermore, large-scale studies are necessary to test the convergent validity compared to traditional biomarkers.

## Author contributions

All authors listed have made a substantial, direct, and intellectual contribution to the work and approved it for publication.
